# Redundant Design in Interdependent Networks

**DOI:** 10.1371/journal.pone.0164777

**Published:** 2016-10-20

**Authors:** Lijun Liu, Yongfeng Yin, Zenghu Zhang, Yashwant K. Malaiya

**Affiliations:** 1 School of Reliability and System Engineering, Beihang University, Beijing, China; 2 Computer Science Department, Colorado State University, Fort Collins, CO, United States of America; Semmelweis University, HUNGARY

## Abstract

Modern infrastructure networks are often coupled together and thus could be modeled as interdependent networks. Overload and interdependent effect make interdependent networks more fragile when suffering from attacks. Existing research has primarily concentrated on the cascading failure process of interdependent networks without load, or the robustness of isolated network with load. Only limited research has been done on the cascading failure process caused by overload in interdependent networks. Redundant design is a primary approach to enhance the reliability and robustness of the system. In this paper, we propose two redundant methods, node back-up and dependency redundancy, and the experiment results indicate that two measures are effective and costless. Two detailed models about redundant design are introduced based on the non-linear load-capacity model. Based on the attributes and historical failure distribution of nodes, we introduce three static selecting strategies-Random-based, Degree-based, Initial load-based and a dynamic strategy-HFD (historical failure distribution) to identify which nodes could have a back-up with priority. In addition, we consider the cost and efficiency of different redundant proportions to determine the best proportion with maximal enhancement and minimal cost. Experiments on interdependent networks demonstrate that the combination of HFD and dependency redundancy is an effective and preferred measure to implement redundant design on interdependent networks. The results suggest that the redundant design proposed in this paper can permit construction of highly robust interactive networked systems.

## Introduction

With the rapid development in modern information and energy technologies, infrastructure networks have become larger and more complex, and show an interdependent relationship between them because of functional dependence. For example, electricity system and communication system are highly coupled with together, where communication system relies on the electricity system to provide electricity, and electricity system needs communication system to provide control[1].

Compared with an isolated network, interdependent networks are prone to be more susceptible to attacks (random or intentional attack) because of coupling between networks [1–3]. Interdependent network has two different types of links, connectivity link and dependency link. Connectivity link is the connection of paths in isolated network whereas the dependency link reflects the coupling between two isolated networks. If a node in network *N*_*A*_ failed and be disconnected from network *N*_*A*_, which will lead to the failure of another node in another network *N*_*B*_ and may result in a further failure of network *N*_*A*_. The process described above occurs recursively, which may lead to a complete collapse of the interdependent systems.

Robust analysis of interdependent network and designing anti-vulnerable topology have received considerable attention in recent years. Buldyrev[1] firstly constructed an interdependent network model and defined the failure mode of network. He used the generation function and percolation theory to derive a percolation value and performed experiments on three interdependent networks (ER-ER, SF-SF, and ER-SF) to verify the theoretical value. After this pioneering study, researchers have investigated methods to enhance the robustness of interdependent networks. A typical model, introduced by Parshani[2], through reducing coupled strength by removing a part of dependency links. Furthermore, the results demonstrated that robustness of interdependent network had considerably increased by decoupling 40% of nodes. Another method is generating autonomy nodes that would not be non-functional under attack. The experiment found that interdependent networks will maintain their function by generating 10% autonomy nodes. Gong[4] introduced a technique to protect certain key nodes, in which 5% of key nodes can be determined using six strategies (random, degree, betweenness centrality, leader-rank, local and page-rank) and protected them from failure when its coupled nodes collapsed. Hongshen[5] presented a defensive measure in which removing certain nodes with lower degree in interdependent networks to suppress cascading failure process.

Based on a comprehensive analysis of the above studies, we determined that initial studies on the robustness of interdependent networks mainly focus on isolated network with a free load[6–8]. However, in reality, most systems have a load, e.g., electricity network, transportation network, etc. The cascading failure of these networks are primarily caused by overload[9], such as a traffic jam or congestion in communication system. Researches on overload failure model primarily focused on isolated network and rarely addressed interdependent networks[10, 11]. Fei Tan et al[12] introduced three coupling preference (assortative, disassortative and random coupling) in interconnected networks, and determined assortative coupling had better advantages in enhancing the robustness of interconnected networks. After a node fails, its load will redistribute to other nodes. In conclusion, there are two types of redistribution strategies: local distribution and global distribution. In local distribution model, the load of failure nodes would redistribute to its adjacent nodes, such as sandpile model introduced by Brummitt et al[13]; however, the load of remaining nodes are dependent on the betweenness centrality of nodes in updated network topology in global distribution model[12, 14].

Studies on cascading failure process of interdependent networks caused by overload are limted and incomprehensive[14]. The hypothesis in the model introduced by Fei Tan et al[12] is that two isolated networks are homogeneous, and load of failure nodes would be redistributed between two networks, which is not suitable for interdependent networks. Brummitt et al[15] assumed that load of failure nodes would only redistribute to its adjacent nodes. The protecting strategy presented by Hongsheng[5] is plausible but in reality difficult to implement because cascading process is extremely fast, and the interval time is too short to use this method. The main reason of nodes failed is interdependent failure or overload. Previous studies, such as the autonomous node[16] generation, focused on reducing the scope of interdependent failures. Thus, this study aims at minimizing the scope of overload failure.

Redundant design, which is an important measure used to improve the reliability of the system, is used more commonly in reliability design[17, 18]. Thus, in this paper, to suppress overload failure of nodes, we introduce a novel method-redundant design in construction of interdependent networks and analyze different effects of two kinds of redundant strategies. The remainder of this paper can be described as follows: section 2 presents two redundant design models; section 3 discusses the results of simulation; and section 4 gives the conclusion.

## Methods Study

### Cascading process

**Fig 1:**
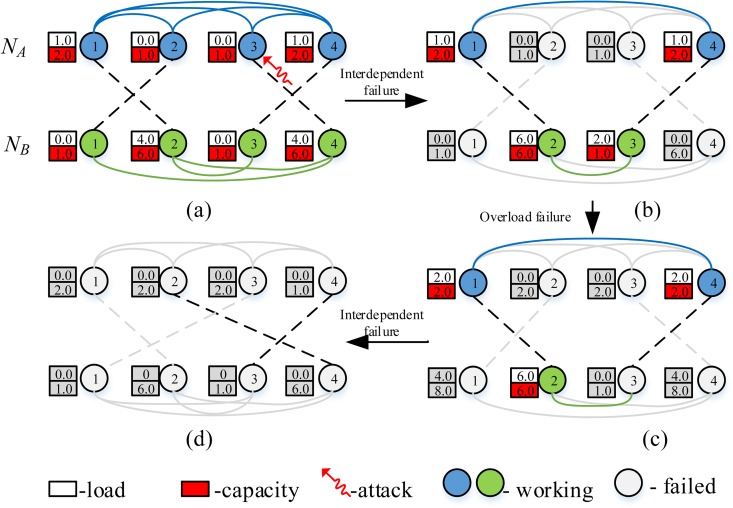
Cascading failure of interdependent network with traffic.

Without loss of generality, two isolated networks (labeled *N*_*A*_ and *N*_*B*_) with the same nodes (*N*_*A*_ = *N*_*B*_ = *N* = 300) are investigated. The typical topology of interdependent networks is illustrated in Fig 1. Connectivity links (blue or green solid line) in two isolated networks could transfer traffic but dependent links (black dash line) could not. Dependent links between two isolated networks are bidirectional and only provide functional and logical connectivity between two networks. If a node in network *N*_*A*_ failed, which would lead to another node in network *N*_*B*_ collapsed because of functional dependency, which means interdependent failure.

A isolated network is a pair of (*V*, *E*), with *V* the set of all nodes {*v*_1_, *v*_2_, …, *v*_n_} and *E* the set of all edges {*e*_1_, *e*_2_, …, *e*_n_}. A path *P*(*v*_*i*_, *v*_*k*_) between node *v*_*i*_ and *v*_*k*_ is a subset of consecutive edges, i.e. *P*(*v*_*i*_, *v*_*k*_) = {*e*_1_, *e*_2_, …, *e*_*j*_}∈*E*. The length |*P*(*v*_*i*_, *v*_*k*_)| of a path *P*(*v*_*i*_, *v*_*k*_) is given by the number of edges in it. There are several paths from node *v*_*i*_ to *v*_*k*_, and the distance *d*(*v*_*i*_, *v*_*k*_) between *v*_*i*_ and *v*_*k*_ is defined as the shortest path(i.e. minimal length of |*P*(*v*_*i*_, *v*_*k*_)|). From the definition of *d*(*v*_*i*_, *v*_*k*_), perhaps several path *P*(*v*_*i*_, *v*_*k*_) exist and whose length |*P*(*v*_*i*_, *v*_*k*_)| are all equal to *d*(*v*_*i*_, *v*_*k*_).

Each node *v*_*j*_ in interdependent network is allocated load *L*^*t*^(*v*_*j*_) which fluctuates timely with the topology of network. For initial load *L*^*0*^(*v*_*j*_) (*t* = 0) of each node, we can take betweenness centrality to calculate it and its formula is expressed as follows:

$$L^{0}(v_{j})=\sum_{i\neq k=1}^{N}\frac{N(d(v_{i},v_{k}:v_{j}))}{N(d(v_{i},v_{k}))}$$ (1)

Where, d(*v*_*i*_, *v*_*k*_: *v*_*j*_) represents the shortest path *P*(*v*_*i*_, *v*_*k*_) which pass through node *v*_*j*_ and *N*(d(*v*_*i*_, *v*_*k*_: *v*_*j*_)) is the number of d(*v*_*i*_, *v*_*k*_: *v*_*j*_)

The load of node *v*_*j*_ at time *t*, *L*^*t*^(*v*_*j*_), varies with the topology of whole network. After a node failed, its load would delivery to other functional nodes. Here we assume that the load of failed nodes transfer evenly to remaining functional nodes in network which is expressed as follows:

$$L^{t}(v_{j})=L^{t-1}(v_{j})+\frac{\sum L^{t-1}(v_{i})}{\left|V_{functional}\right|},v_{i}\in V_{failed},v_{j}\in V_{functional}$$ (2)

Where, *V*_*functional*_ and *V*_*failed*_ are set of nodes that remain functional at time *t* and failed at time *t*-1, respectively. |*V*_*functional*_| denotes number of node in *V*_*functional*_.

Due to restriction of cost, the capacity of a node is limited. A node’s capacity means the ability to bear load on it mostly. For studying cascading process on complex network with traffic, Motter and Lai[11] proposed that the capacity of node *C*(*v*_*i*_) was proportional to its initial load *L*^*0*^(*v*_*i*_), as showed as follows

$$C(v_{i})=L^{0}(v_{i})(1+\alpha ),i=1,2,...,N$$ (3)

Where, α ≥ 0 is the tolerance coefficient of node *v*_*i*_, and greater of it means node *v*_*i*_ could handle more load on it. However, Kim and Motter[19] found that there was no linear relationship between node’s load and capacity by analyzing four real networks. And Dou B.[20] proposed a non-linear load-capacity model, which seemed more suitable for real system. In this paper, we adopt this model to determine the capacity of nodes, and the model can be described as follows

$$C(v_{i})=L^{0}(v_{i})+\beta {\left(L^{0}(v_{i})\right)}^{\alpha },i=1,2,...,N$$ (4)

Here, α ≥ 0 and β ≥ 0. When α = 1, this model degenerates to ML model.

In addition, the cost of network is depend on nodes capacity, which means that with the increase of α and β, we should allocate more resource on network. In this paper, we define cost of network as follows

$$Cost=\sum_{i=1}^{N}C(v_{i})$$ (5)

And according to the findings of Kim and Motter[19], due to network traffic fluctuations, real systems tend to have larger unoccupied portions of the capacities—smaller load-to-capacity ratios—on network elements with smaller capacities. To evaluate the efficiency of node’s capacity, we propose a modified model based on Kim and Motter and it is described as follows

$$E\_Value=\sum_{i=1}^{N}\frac{L^{n}(v_{i})}{C(v_{i})}/N$$ (6)

Where, *E_Value* and *L*^*n*^(*v*_*i*_) are the efficiency coefficient and load of node *v*_*i*_ when interdependent networks in a stable state, respectively. And if node *v*_*i*_ failed after cascading process, the value of *L*^*n*^(*v*_*i*_)/*C*(*v*_*i*_) is 1. Different from KM model, our modified model make a weighted average of all nodes’ efficiency.

Once the load of a node surpass its capacity, it will lead to overload failure, which is another failure mode of node in interdependent network. Compared to isolated network, interdependent network exhibits more obvious vulnerability[12]. A small amount of nodes in the network are overload or suffering attack, which may cause cascading failure and lead to further damage. Fig 1 depicts an intuitive example for explaining cascading failure process of interdependent networks when experiencing a random attack. In this cascading process, node labeled 3 in network *N*_*A*_ was attacked, which lead to node labeled 4 in *N*_*B*_ failed. Then, we threat node labeled 1 in *N*_*B*_ failed because of out of giant component, which lead to node labeled 2 in *N*_*A*_ collapsed. After interdependent failure ending, load of all failed nodes in two networks would evenly be distributed to remaining two nodes (as shown in Fig 1(B)), which lead to node labeled 3 in *N*_*B*_ overload. Failure of node labeled 3 in *N*_*B*_ causes a brandnew interdependent failure, resulting a complete breakdown of whole networks (as shown in Fig 1(D)).

Previous studies proposed that the relative value of average network size after the cascading failure process can be selected to evaluate the robustness of interdependent networks under random/intentional attack[1, 4, 14]. The relative value of average network size after cascading failure is defined as follows

$$G=(S^{'}(N_{A})+S^{'}(N_{B}))/2N$$ (7)

Where, *S*′(*N*_*A*_) and *S*′(*N*_*B*_) stand for the number of node in *N*_*A*_ and *N*_*B*_ after cascading process, respectively. To analyze the failure distribution of cascading failure process, we also focus on number of failed nodes caused by interdependency and overload, respectively.

**Fig 2:**
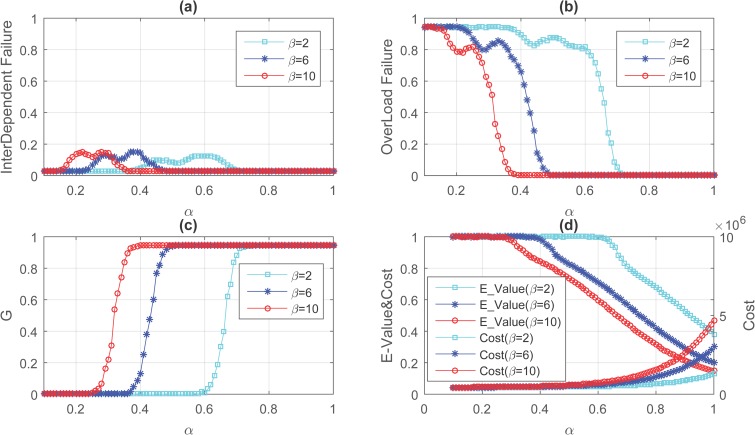
Cascading process on ER-ER interdependent network with node number *N* = 300 and <k> = 6. (Underlying data are in Text 8-Text 13 in S2 File).

We firstly construct two Erdős-Rényi networks according to the literature[21], whose degree distribution obeys the Poisson distribution and parameter *N*_*A*_ = *N*_*B*_ = *N* = 300 and average degree <k_A_> = <k_B_> = <k> = 6. Then constructing an ER-ER interdependent network by coupling two Erdős-Rényi networks. We randomly attack a proportion, 5%, of nodes in each network and follow the iterative process of cascading failure, and do simulation over 50000 times on different value of α and β. The simulation results of cascading failure process with diverse α and β are demonstrated in Fig 2. Obviously, the behavior of *G* is characteristic of a first-order phase transition in ER-ER interdependent network.

In ER-ER interdependent network, for a certain β, a lower limit *α*_*L*_ and upper limit *α*_*U*_ are determined. When α ≤ *α*_*L*_, whole networks collapsed and all nodes failed caused by interdependency or overload and overload failed nodes account for most. While α > *α*_*U*_, despite those nodes be attacked at first, no more nodes in networks failed. [*α*_*L*_,*α*_*U*_] is a phase transition interval and during this interval, the value of *G* increases rapidly with the growing of α and overload failed nodes decreases quickly at the same time and the cascading failure process come to its end in a short time.

**Fig 3:**
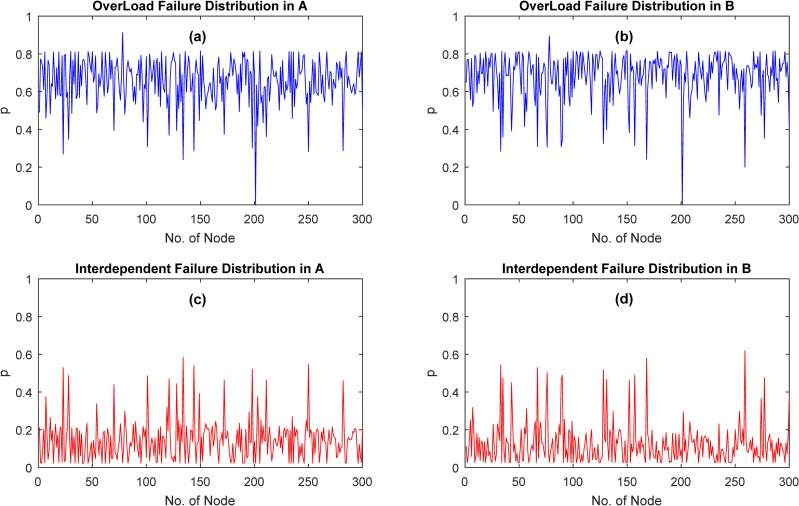
Failure distribution of ER-ER interdependent network with β = 6 and α = 0.4 over 100000 simulation times. (Underlying data are in Text 14-Text 17 in S3 File).

According to the results of Fig 2(A) and Fig 2(B), we can conclude that overload failed nodes have been accounted for 70% when β = 6 and α = 0.4, the number of interdependent failed nodes is large simultaneously. Thus, we fix β = 6 and α = 0.4 and do simulation over 100000 times, the results are shown in Fig 3. We have counted the failed time for each node over 100000 times, including interdependent failure and overload failure. Interesting, when β = 6 and α = 0.4, the frequency of most nodes in *N*_*A*_ or *N*_*B*_ failed because of overload is about 70%, which is the proportion of overload failed nodes, and several nodes failed with a slightly lower frequency compared to most nodes. In contrast, some nodes failed due to interdependency with a higher frequency compared to other nodes.

### Redundant design

As described in Fig 2(C), we can increase the value of α and β to reduce the size of cascade avalanche, which also improving the cost of networks and reducing the value of *E_Value* at the same time. And perhaps it is a troublesome problem to enhance the capacity of node in the current technology level. Thus, it is a worth considering question that how we can utilize existing resources to heighten the robustness of whole networks. The redundancy allocation is one of the most advantageous methods to optimize system reliability [22–25]. For some key components in system, designer will allocate more resources to those to enhance their ability to handle catastrophic events. Because of aging or suffering attack, a component failed and it will still remain be functional if it has a backup and it would collapse only its back-up failed. With this measure, the failure rate of component in systems would decrease drastically and reliability of whole system enhances simultaneously.

### Nodes back-up

**Fig 4:**
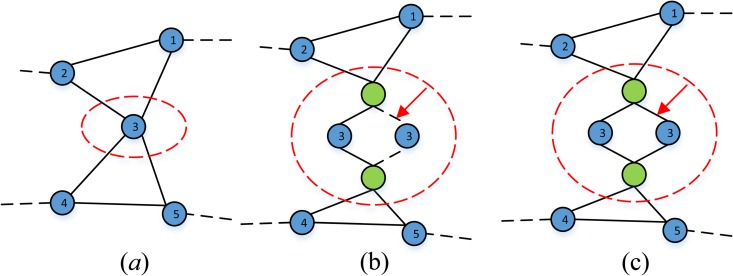
Nodes back-up model.

To suppress overload failure of nodes and inspired by redundant design in reliability domain, this paper proposes a redundant design to some nodes in interdependent network. As shown in Fig 4(A), compared to other four nodes (labeled 1, 2, 4 and 5), node labeled 3 is determined with a key node according to the value of betweenness centrality, its failure would break down four nodes to two disconnected parts and lead to whole network topology collapsed accordingly. If we make an addition of node labeled 3 and two joint nodes (filled green) whose reliability value is 1, and two joint nodes could switch on successfully to connect back-up if necessary, which would reduce the failure rate of node labeled 3 and maintain whole network connected like before.

In this model, we introduce the concept of node unit *U*(*v*_*i*_) which represents set of nodes with same function (red dashed ellipse in Fig 4) and define *U*(*v*_*i*_) as follows:

$$U(v_{i})=\underset{n}{\underset{︸}{\left\{v_{i}\cdots v_{i}\right\}}},n\in \mathbb{N},i=1,\cdots ,N$$ (8)

where *n* represents redundant level and it means that node *v*_*i*_ has no back-up if *n* = 1;

Then, we can determine the capacity of node unit *U*(*v*_*i*_), C(*U*(*v*_*i*_)), according to our redundant model. Clearly C(*U*(*v*_*i*_)) is the sum of capacity of all node

$$C(U(v_{i}))=n\times C(v_{i}),n\in \mathbb{N},i=1,\cdots ,N$$ (9)

And the cost of network after redundant design is increment to

$$Cost=\sum_{i=1}^{N}C(U(v_{i}))$$ (10)

We define initial node as main node and it should be activate firstly. If main node could bear load that passes through on it, it is unnecessary to activate its back-up, and back-up would be in work immediately to balance load once main node couldn’t handle all load on it. With this mechanism, the value of *E_Value* of main node and back-up which has been activated will maintain a larger level and node unit *U*(*v*_*i*_) would not collapse at the same time. However, the reliability and successful rate of activating back-up are two important question. This paper assumes that joint nodes are reliable and the successful rate of activating back-up is always equal to 1.

Determining which nodes would have a back-up is really important, which directly effects the result of implementation. In this paper, we introduce four kinds of select strategies, Random-based, Degree-based, Initial Load-based, and HFD-based (historical failure distribution).

**Fig 5:**
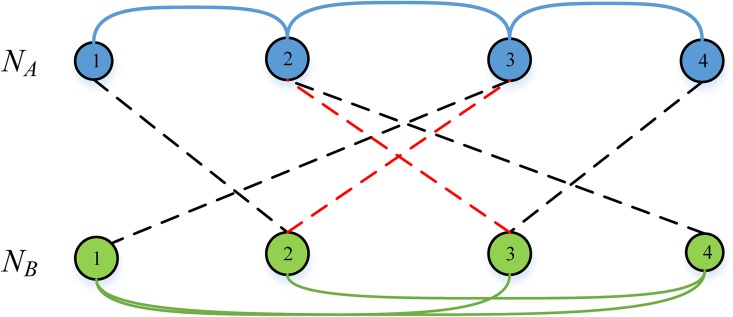
Dependency redundancy model.

The first three strategies, mainly based on static attributes of node, can be classified as static method. Random-based, which means we randomly choose some nodes in network. Degree and Initial Load-based, which according to the ranking of degree and initial load value of nodes and then pick those nodes listed in the forefront with a proportion. HFD (as shown in Fig 4 and Fig 5), which based on the simulation results of cascading failure over large-scale simulation times, is classified as a dynamic method and we can implement redundant design more accurately with this method. In HFD, we select those nodes with higher frequency of overload or interdependent failure compared to other nodes.

### Dependency redundancy

To reduce the number of nodes caused by interdependent failure, we introduce a model named dependency redundancy. According to the failure mode of interdependent network, a node failed in *N*_*A*_ will lead to another node in *N*_*B*_ collapsed, which may cause an iterative cascading process. Because each node in *N*_*A*_ only can find a node in *N*_*B*_ to be coupled with, in other word, only a node in *N*_*B*_ could provide functional or logical dependency to node in *N*_*A*_.

As shown in Fig 5, network *N*_*A*_ (whose nodes filled blue) and network *N*_*B*_ (whose nodes filled green) are coupled together, and each node is coupled with a certain node in another network through dependency link (black dotted line). Before adopting dependency redundancy, if node labeled 1 in *N*_*A*_ suffering from attack or overload, which would lead to failure of node labeled 2 in *N*_*B*_. Here we randomly add two dependency links (red dotted line) in interdependent networks. If the same thing happened (node labeled 1 in *N*_*A*_ failed), node labeled 2 in *N*_*B*_ would not collapse because node labeled 3 in *N*_*A*_ could provide functional support. Similarly, failure of node labeled 4 in *N*_*A*_ would not result in node labeled 3 disconnected from *N*_*B*_.

Adding some dependent links in networks would increase cost of design, which is a question that should be studied. In this paper, we assume that cost of dependent link is depend on capacity of two nodes, and we defined it as follows

$$C(l(v_{i},v_{j}))=max\{C(v_{i}),C(v_{j})\},v_{i}\in V(N_{A}),v_{j}\in V(N_{B})$$ (11)

Where, *C*(*l*(*v*_*i*_,*v*_*j*_)) means the cost of adding a dependent links between node *v*_*i*_ and node *v*_*j*_.

### Simulation algorithm

To study the impact of redundant design in interdependent networks, we have applied the following simple simulated algorithm. Firstly we give some necessary definitions about some symbols and operations.

        *AM* (*N*_*A*_), *AM* (*N*_*B*_)←*adjacency matrix of N*_*A*_ and *N*_*B*_, respectively

        *CN*(*N*_*A*_), *CN*(*N*_*B*_) ← 1×*N* vector (out of order), node pair (*CN*(*N*_*A*_)(1,*i*),*CN*(*N*_*B*_)(1,*i*)) (*i* = 1:*N*) represents that they are interdependent in networks

        *CN* (*CN*(*N*_*A*_), *CN*(*N*_*B*_))←interdependence relation between *N*_*A*_ and *N*_*B*_

        *AP*←attack proportion

        *V*_*A*_←attack nodes

        $V_{IF}^{t}(N_{A})$, $V_{IF}^{t}(N_{B})\leftarrow$set of failed nodes caused by dependency at time *t* in *N*_*A*_ and *N*_*B*_, respectively

        $V_{OF}^{t}(N_{A})$, $V_{OF}^{t}(N_{B})\leftarrow$set of failed nodes caused by overload at time *t* in *N*_*A*_ and *N*_*B*_, respectively

        $V_{GF}^{t}(N_{A})$, $V_{GF}^{t}(N_{B})\leftarrow$set of failed nodes because of out of giant component at time *t* in *N*_*A*_ and *N*_*B*_, respectively

        *V*(*N*_*A*_), *V*(*N*_*B*_)←set of failed nodes during cascading process in *N*_*A*_ and *N*_*B*_, respectively

        *V*(*N*_*A*_)⨂*CN* (*CN*(*N*_*A*_), *CN*(*N*_*B*_))←failed nodes in *N*_*B*_ because of coupled with *V*(*N*_*A*_)

        *V*(*N*_*B*_)⨂*CN* (*CN*(*N*_*A*_), *CN*(*N*_*B*_))←failed nodes in *N*_*A*_ because of coupled with *V*(*N*_*B*_)

        The pseudo-code of simulated algorithm is as follows

Program: Initial Configuration Module

        *N*_*A*_ = (*V*, *E*, *AM* (*N*_*A*_))←generating *N*_*A*_.

        *N*_*B*_ = (*V*, *E*, *AM* (*N*_*B*_))←generating *N*_*B*_.

        *CN*(*N*_*A*_), *CN*(*N*_*B*_) ←generating coupling nodes pair

        *L*^*0*^(*v*_*i*_) ←calculating initial load of nodes in *N*_*A*_ and *N*_*B*_

        *C*(*v*_*i*_) ←calculating capacity of nodes in *N*_*A*_ and *N*_*B*_

End Initial Configuration Module

Program: Cascading Process Module

        *V*_*A*_ = *AP* × 2*N*,

        *V*(*N*_*A*_) = *V*_*A*_(1:*length*(*V*_*A*_)/2)

        *V*(*N*_*B*_) = *V*_*A*_(*length*(*V*_*A*_)/2 + 1:*length*(*V*_*A*_))

        $V_{IF}^{t}(N_{A})$ = *V*(*N*_*B*_)⨂*CN* (*CN*(*N*_*A*_), *CN*(*N*_*B*_))

        $V_{IF}^{t}(N_{B})$ = *V*(*N*_*A*_)⨂*CN* (*CN*(*N*_*B*_), *CN*(*N*_*A*_))

        *V*(*N*_*A*_) = *V*(*N*_*A*_)+$V_{IF}^{t}(N_{A})$

        *V*(*N*_*B*_) = *V*(*N*_*B*_)+$V_{IF}^{t}(N_{B})$

        Load_A = 0

        Load_B = 0

        *t* = 0

        **Do While** $V_{IF}^{t}(N_{A})\neq \emptyset \&\&V_{IF}^{t}(N_{B})\neq \emptyset \&\&V_{OF}^{t}(N_{A})\neq \emptyset {\&\&V}_{OF}^{t}(N_{B})\neq \emptyset$

                **Do While** $V_{IF}^{t}(N_{A})\neq \emptyset \&\&V_{IF}^{t}(N_{B})\neq \emptyset \&\&V_{GF}^{t}(N_{A})\neq \emptyset \&\&V_{GF}^{t}(N_{B})\neq \emptyset$

                Remove {$V_{IF}^{t}(N_{A}),V_{IF}^{t}(N_{B}),V_{GF}^{t}(N_{A}),V_{GF}^{t}(N_{B})$} and links that connected with those

                Nodes, and update $V_{GF}^{t}(N_{A})\;and\;V_{GF}^{t}(N_{B})$

                $V_{IF}^{t}(N_{A})=V_{GF}^{t}(N_{B})⨂$*CN* (*CN*(*N*_*A*_), *CN*(*N*_*B*_))

                $V_{IF}^{t}(N_{B})=V_{GF}^{t}(N_{A})⨂$*CN* (*CN*(*N*_*B*_), *CN*(*N*_*A*_))

                *V*(*N*_*A*_) = *V*(*N*_*A*_)+$V_{IF}^{t}(N_{A})+V_{GF}^{t}(N_{A})$

                *V*(*N*_*B*_) = *V*(*N*_*B*_)+$V_{IF}^{t}(N_{B})+V_{GF}^{t}(N_{B})$

                Load_A = Load_A+$\sum_{v_{j}\in \{V_{IF}^{t}(N_{A}),V_{GF}^{t}(N_{A})\}}L^{t}(v_{j})$

                Load_B = Load_B+$\sum_{v_{j}\in \{V_{IF}^{t}(N_{B}),V_{GF}^{t}(N_{B})\}}L^{t}(v_{j})$

                **EndWhile**

                *t = t+*1

                *V*_*t*_ = *V*(*N*_*A*_)∩ *V* in *N*_*A*_

                **For** i = 1 *to length*(*V*_*t*_)

                        *L*^*t*^(*v*_*i*_) = *L*^*t*−1^(*v*_*i*_) + Load_A/ *length*(*V*_*t*_)

                        **If** *L*^*t*^(*v*_*i*_) > *C*(*v*_*i*_)

                                $V_{OF}^{t}(N_{A})=V_{OF}^{t}(N_{A})+v_{i}$

                        **EndIf**

                **EndFor**

                *V*_*t*_ = *V*(*N*_*B*_)∩ *V* in *N*_*B*_

                **For** i = 1 *to length*(*V*_*t*_)

                        *L*^*t*^(*v*_*i*_) = *L*^*t*−1^(*v*_*i*_) + Load_B/ *length*(*V*_*t*_)

                        **If** *L*^*t*^(*v*_*i*_) > *C*(*v*_*i*_)

                                $V_{OF}^{t}(N_{B})=V_{OF}^{t}(N_{B})+v_{i}$

                        **EndIf**

                **EndFor**

        **EndWhile**

## Results

To analyze the role of the redundant design, we have applied two redundant design methods on ER-ER interdependent networks. For a better simulation result, each redundant proportion is simulated over 5000 times.

**Fig 6:**
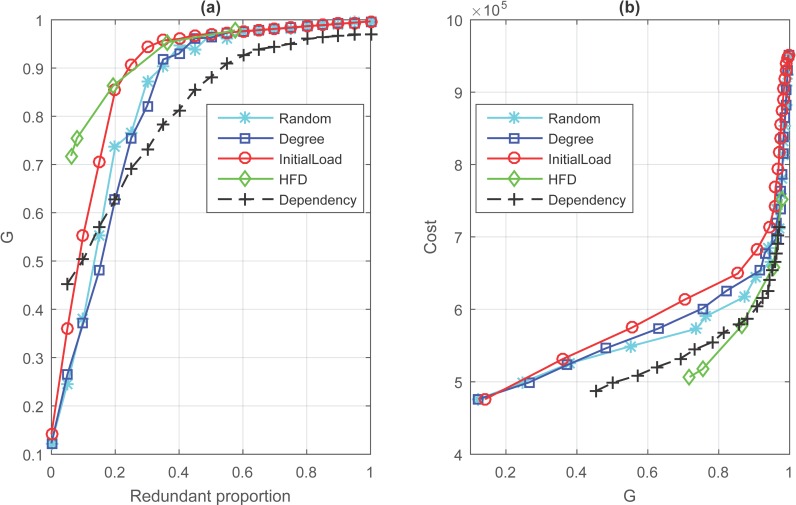
Simulation results of four node back-up strategies and dependency redundancy. (Underlying data are in Text 18-Text 32 in S4 File).

The simulation results of cascading process with different proportion and four kinds of node back-up strategies are demonstrated in Fig 6. The simulations parameters are *N*_*A*_ = *N*_*B*_ = 300, <*k*_*A*_> = <*k*_*B*_> = 6, β = 6, α = 0.4. If we don’t consider the cost of redundant design, obviously, HFD back-up strategy is a preferred method on the whole when the redundant proportion is a constant. As pictured in Fig 6(A), when 5% of nodes in whole network have back-up, it would get almost 60% of enhancement on G compared to no redundant proportion, which means we could maintain 70% of nodes when 5% of nodes have a back-up. In comparison, degree-based and random-based back-up strategy have similar result and perform worst. But once we take consideration of the cost of redundant design, the results will be a big difference. From Fig 6(B), we note that HFD is still the best way and dependency redundancy has gradually show its advantage, while initial load-based back-up strategy becomes the worst because it seemly costly. And we noticed that the cost of redundant design has a shape rise when we want to maintain 90% of initial network survival.

HFD, a new and distinct method that based on the distribution of failed nodes, performs better with a low cost and high efficiency. In Fig 3, it is indicated that most nodes have a higher frequency of overload failure, which means it is unwise to select nodes based on this results. While the distribution of interdependent failure is different from overload failure-several nodes have a higher frequency of interdependent failure compared to the rest. If we control the failure of nodes which coupled with those nodes with higher frequency of interdependent failure, then interdependent failure would eliminate a lot. For example, when 8% of nodes have a back-up based on historical failure distribution, which will keep 78% of nodes in initial network survival with the least cost. And from Fig 6, it illustrates that HFD is the most effective and costless method compared to others. In HFD-based method, the number of nodes that have a back-up are determined by the results of interdependent failure. Thus only a part of nodes can be determined by HFD, which could not protect whole network from failure.

The simulation results of dependency redundancy are also demonstrated in Fig 6, which illustrates that dependency redundancy is not an ideal method to improve the value of *G* compared with other four node back-up strategies when robustness is the first consideration. However, if we consider cost of implementation, dependency redundancy has more advantages to others. And we notice that it performs similar with HFD, and perhaps combination these two measures will get unexpected results.

**Fig 7:**
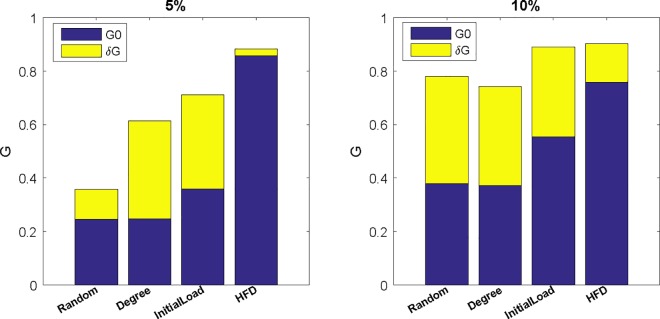
Effect on *G* of combination of four node back-up strategies and dependency redundancy.

**Fig 8:**
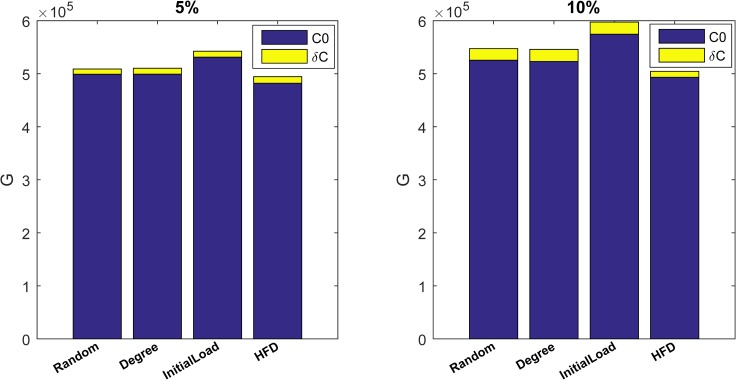
Effect on *Cost* of combination of four node back-up strategies and dependency redundancy.

To illustrate this hypothesis, we combine two redundant measures and the results are demonstrated in Fig 7 and Fig 8. In the experiment, 5% and 10% of nodes are selected based four measures, respectively, meanwhile 5% and 10% of dependency links are randomly constructed. In Fig 7 and Fig 8, *G*0 and *C*0 stand for value of *G* and *Cost* after cascading process when only 5% of nodes have a back-up, respectively, while δ*G* and δ*C* are the increment of *G* and *Cost* from 5% and 10% dependency redundancy. From Fig 7 and Fig 8, it is clearly that adding a small proportion of dependent links will obtain a significant enhancement on *G* while with a tiny cost incrementation. Thus through a combination of node back-up and dependency redundancy, we can construct more robust interdependent network with lower cost and resource.

Thus, in ER-ER interdependent network, HFD, a dynamic method, is the first choice to determine which nodes that would have back-up. And dependency redundancy is also suggested with minimal cost when no back-up could deploy. If possible, combination of two measures is the most effective and costless scheme.

## Conclusion

In this paper, we have studied potential cascading failures of interdependent networks with traffic under random attacks when system incorporates redundant design.

Based on the non-linear load-capacity model, we can enhance the invulnerability of interdependent networks under random attack through following two methods:

1. If technology allows, we can increase value of α and β, and a phase transition interval [*α*_*L*_,*α*_*U*_] for each β could help us to determine the most suitable value of α;
2. If conditions are limited, the redundant design proposed in this paper could be a nice suggestion to be considered.

In ER-ER interdependent networks, the experimental results indicated that HFD is a preferred measure to decide which nodes could have a back-up with lower cost. While we also could select nodes based on its initial load if robust is more important than cost. And if cost of design is limited and no more back-up could be deployed, dependency redundancy may be an ideal way with a minimal cost to get higher *G* value.

If we want to construct a more robust interdependent system, node back-up and dependency redundancy should be adopted in the beginning. Combination of two kinds of measures is attractive by its higher efficiency and lower cost, which could suppress overload failure more accurately and reduce the scope of interdependent failure.

Therefore, the proposed preventive measure for enhancing the survivability of interdependent networks can be used effectively when constructing robust networked systems. This approach provides a practicable technical method to design and optimize these networks with lower cost. And combination of node back-up and dependency redundancy could be studied deeply in the future.

## Supporting Information

S1 File Text 1: Adjacency matrix of network A. Text 2: Adjacency matrix of network B. Text 3: Initial load of each nodes in network A. Text 4: Initial load of each nodes in network B. Text 5: Coupling between two networks. Text 6: Initial degree of each nodes in network A. Text 7: Initial degree of each nodes in network B.
(ZIP)

S2 File Text 8: Interdependent failed nodes at different **α** and **β** duiring 100 simulation times of Network A. Text 9: Interdependent failed nodes at different **α** and **β** duiring 100 simulation times of Network B. Text 10: Nodes that are not belong to gaint component in network A at different **α** and **β** duiring 100 simulation times of Network A. Text 11: Nodes that are not belong to gaint component in network B at different **α** and **β** duiring 100 simulation times of Network B. Text 12: Overload failed nodes at different **α** and **β** duiring 100 simulation times of Network A. Text 13: Overload failed nodes at different **α** and **β** duiring 100 simulation times of Network B.
(ZIP)

S3 File Text 14: Interdependent failed nodes accounts in network A over 100000 simulations times. Text 15: Interdependent failed nodes accounts in network B over 100000 simulations times. Text 16: Overload failed nodes accounts in network A over 100000 simulations times. Text 17: Overload failed nodes accounts in network B over 100000 simulations times.
(ZIP)

S4 File Text 18: Redundant proportion and nodes in network A with Random-based node back-up strategy. Text 19: Redundant proportion and nodes in network B with Random-based node back-up strategy. Text 20: Failed nodes in different redundant proportion in network A with Random-based node back-up strategy. Text 21: Redundant proportion and nodes in network A with Degree-based node back-up strategy. Text 22: Redundant proportion and nodes in network B with Degree-based node back-up strategy. Text 23: Failed nodes in different redundant proportion in network A with Degree -based node back-up strategy. Text 24: Redundant proportion and nodes in network A with InitialLoad-based node back-up strategy. Text 25: Redundant proportion and nodes in network B with InitialLoad-based node back-up strategy. Text 26: Failed nodes in different redundant proportion in network A with InitialLoad-based node back-up strategy. Text 27: Redundant proportion and nodes in network A with HFD-based node back-up strategy. Text 28: Redundant proportion and nodes in network B with HFD-based node back-up strategy. Text 29: Failed nodes in different redundant proportion in network A with HFD-based node back-up strategy. Text 30: Redundant proportion and nodes in network A with Dependency redundancy. Text 31: Redundant proportion and nodes in network B with Dependency redundancy. Text 32: Failed nodes in different redundant proportion in network A with Dependency redundancy.
(ZIP)
